# Potential clinical biomarkers and perspectives in diabetic cardiomyopathy

**DOI:** 10.1186/s13098-023-00998-y

**Published:** 2023-03-04

**Authors:** Jianxin Deng, Fang Yan, Jinglun Tian, Aijun Qiao, Dewen Yan

**Affiliations:** 1 Department of Endocrinology, Shenzhen Second People’s Hospital, the First Affiliated Hospital of Shenzhen University, Health Science Center of Shenzhen University, Shenzhen Clinical Research Center for Metabolic Diseases, No. 3002, Sungang West Road, Futian District, Shenzhen, 518035 Guangdong Province China; 2 Geriatric Diseases Institute of Chengdu, Center for Medicine Research and Translation, Chengdu Fifth People’s Hospital, Chengdu, 611137 Sichuan Province China; 3 Department of Geriatrics, the Traditional Chinese Medicine Hospital of Wenjiang District, Chengdu, 611130 China; 4 Zhongshan Institute for Drug Discovery, Shanghai Institute of Materia Medica, Chinese Academy of Sciences, Zhongshan, 528400 Guangdong Province China; 5 Shanghai Institute of Materia Medica, Chinese Academy of Sciences, 555 Zu Chong Zhi Road, Shanghai, 201203 China

**Keywords:** Diabetic cardiomyopathy, Biomarkers, Apelin, Irisin, Galactin-3, NLRP3 inflammasome

## Abstract

Diabetic cardiomyopathy (DCM) is a serious cardiovascular complication and the leading cause of death in diabetic patients. Patients typically do not experience any symptoms and have normal systolic and diastolic cardiac functions in the early stages of DCM. Because the majority of cardiac tissue has already been destroyed by the time DCM is detected, research must be conducted on biomarkers for early DCM, early diagnosis of DCM patients, and early symptomatic management to minimize mortality rates among DCM patients. Most of the existing implemented clinical markers are not very specific for DCM, especially in the early stages of DCM. Recent studies have shown that a number of new novel markers, such as galactin-3 (Gal-3), adiponectin (APN), and irisin, have significant changes in the clinical course of the various stages of DCM, suggesting that we may have a positive effect on the identification of DCM. As a summary of the current state of knowledge regarding DCM biomarkers, this review aims to inspire new ideas for identifying clinical markers and related pathophysiologic mechanisms that could be used in the early diagnosis and treatment of DCM.

## Introduction

Diabetes is a metabolic disease characterized primarily by hyperglycemia. In the twenty-first century, diabetes and its complications have risen to prominence as the most rapidly expanding major public health issue worldwide. It is estimated that the number of adults diagnosed with diabetes has tripled in the last 20 years due to factors such as rising incomes, shifting dietary habits, and an aging population. According to the latest International Diabetes Federation (IDF) projections, the prevalence of diabetes in adults is currently around 10.2%, and the global diabetes population is expected to reach 578 million by 2035, the global diabetes population is expected to reach 700 million by 2045 [1]. The prevalence of diabetes in adults is currently around 10.2 percent. The cost of diabetes care and complications is high for the individual, their loved ones, and the community as a whole. Major complications in diabetic patients include cardiovascular events like diabetic cardiomyopathy (DCM), which is the leading cause of death among diabetics [2]. Rubler et al. proposed the concept of DCM in 1972, after describing the occurrence of heart failure symptoms in diabetic patients who did not have coronary or valvular disease. According to the European Society of Cardiology and the European Diabetes Association (ESCEDA), DCM is defined as left ventricular diastolic dysfunction (tissue Doppler, E/E’ ratio ≥ 15) in diabetic patients without hypertension (HT), ischemic heart disease, or other structural heart diseases [3]. DCM develops gradually from asymptomatic myocardial diastolic dysfunction to symptomatic congestive heart failure (HF), putting patients’ lives in jeopardy.

## Epidemiology of DCM

It is estimated that between 70 and 80% of all deaths from diabetes are caused by cardiovascular problems. Excluding confounding variables like age, the Framingham Heart Study indicated that the risk of final cardiovascular morbidity showed that the risk of cardiovascular morbidity increased two-fold in men with diabetes and up to three times in women compared to non-diabetic patients [4]. Patients with diabetes are at a greater risk of developing heart failure (prevalence between 19 and 23%), and vice versa [5–7]. According to echocardiographic studies, 40–60% of asymptomatic diabetic patients have diastolic dysfunction. Patients with diabetes and subclinical diastolic dysfunction had a higher 5-year death risk than those without diabetes and diastolic dysfunction (30.8% vs 12.1%) [8, 9]. In addition, patients with diabetes or HF are mutually reinforcing, people with diabetes being more likely to develop HF and patients with HF being more likely to develop diabetes [10–12]. Diabetic patients often present with diastolic and systolic dysfunction due to left ventricular hypertrophy and wall thickness [13]. The damage to the coronary arteries is also more severe in diabetics, and the risk of damage to numerous vessels at once is higher in diabetics. HF risk is closely correlated with how well glucose levels are maintained over time. The risk of HF increases by 8% for people with type 2 diabetes mellitus (T2DM) for every 1% increase in hemoglobin A1c and 30% for those with type 1 diabetes for every 1% increase in glycosylated hemoglobin [14].

## Mechanisms contributing to the development of DCM

**Fig. 1:**
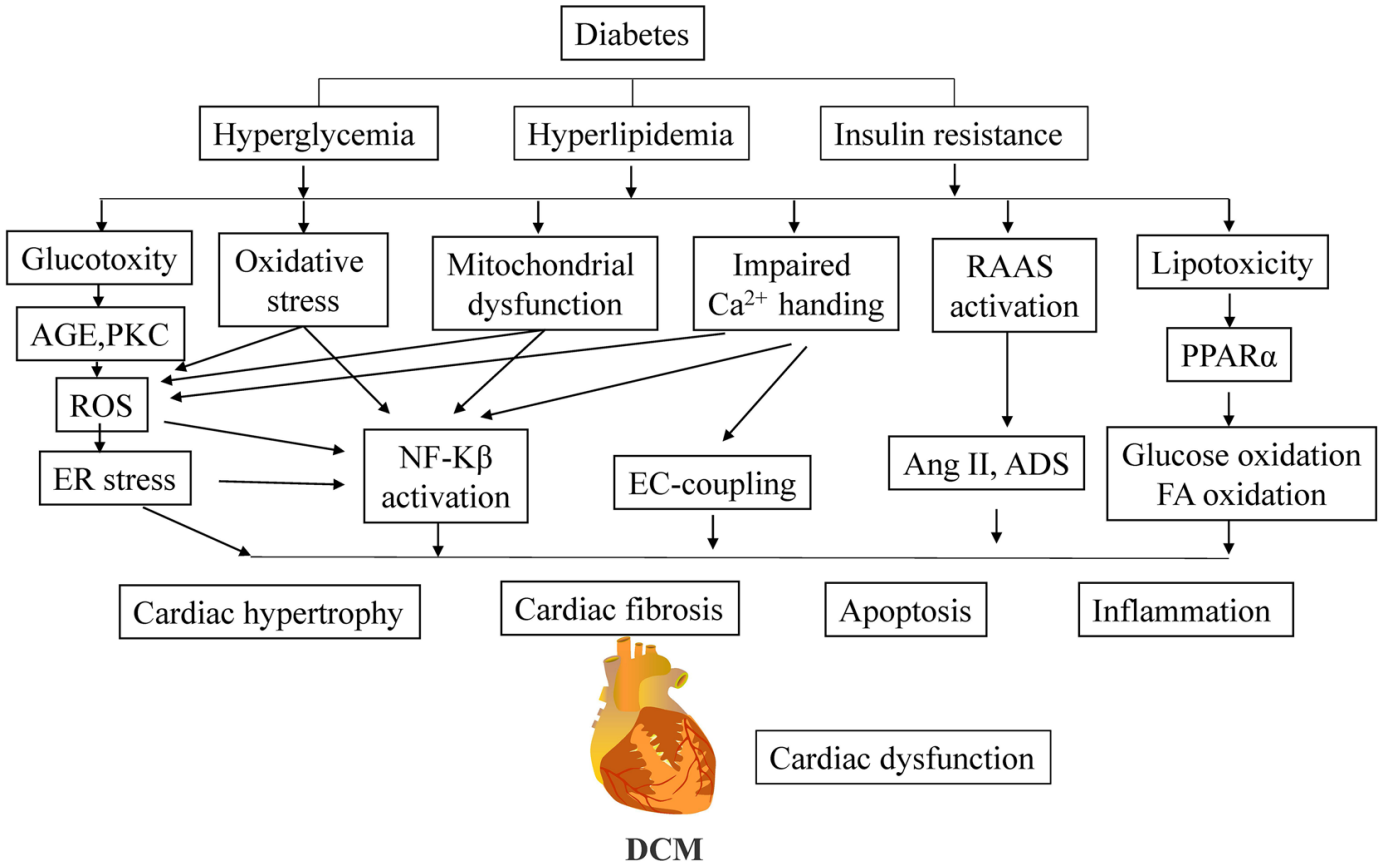
Diabetic cardiomyopathy pathophysiological mechanisms

DCM pathogenesis is complex, with multiple mechanisms involved, including myocardial metabolic disorders, cardiac oxidative stress, inflammatory responses, abnormal Ca^2+^ regulation, and abnormal mitochondrial function (Fig. 1). In brief, (1) hyperglycemia disrupts the metabolism of cardiac cells and damages the heart by increasing reactive oxygen species (ROS), advanced glycosylation end products (AGEs), activating the hexosamine pathway, the polyol pathway, protein kinase C, etc. Increased lipid beta-oxidation, accumulation of triglycerides and free fatty acids in cardiac cells, and subsequent free fatty acid synthesis and ROS production can all contribute to myocardial injury in people with diabetes mellitus. Insulin resistance (IR) is a major risk factor for diabetic cardiovascular problems and is influenced by the activation of the phosphatidylinositol 3-kinase (PI3K)/adenylate kinase (AKT) and mitogen-activated protein kinase (MAPK) signaling pathways. (2) Renin–angiotensin–aldosterone system (RAAS) activation matrix metalloproteinases (MMPs) play a critical role in maintaining extracellular matrix collagen homeostasis for myocardial fibrosis in DCM, and RAAS activation is intimately linked to the progression of HF in diabetes. (3) the major promoters of the exogenous and endogenous apoptotic pathway, caspase-8 and caspase-9, are activated in cardiomyocytes by high glucose (HG) levels. However, the death of cardiomyocytes, caused by caspases-8 and caspases-9, also contributes to the onset of diabetic cardiomyopathy. (4) Abnormal microvascular diastoles, increased permeability of the vessel wall, thickening of the basement membrane, and the progression to DCM; (5) are all effects of vascular endothelial dysfunction. DCM is caused in part by an overreaction to oxidative stress and inflammation. In conclusion, DCM is associated with metabolic abnormalities, cardiac fibrosis, cardiomyocyte apoptosis, microangiopathy, oxidative stress, an inflammatory response, and changes in mitochondrial structure and function.

DCM is a distinct pathophysiological state, and it is challenging to detect DCM patients by routine clinical examination because there are no clinical symptoms or signs in the early stages. Consequently, DCM is typically diagnosed only when some kind of cardiac dysfunction occurs, which can have devastating effects on the treatment of DCM patients. Studies are showing that cytokines (including aggregated serum biomarkers, etc.) change significantly in the beginning stages of DCM development, and these alterations are anticipated to be used for early DCM diagnosis. Therefore, it is crucial to identify high specificity and sensitivity biomarkers for DCM to develop a practical method for early diagnosis. By reviewing the progress of recently studied potential biomarkers, this article aims to provide ideas for the clinical exploration of early diagnostic biomarkers screening for DCM.

### Facators associated with disorders of metabolism

#### Adiponectin (APN)

APN, also known as lipocalin, is an insulin-sensitizing hormone. APN is an adipocyte-secreted endogenous bioactive peptide or protein. Previous research has shown that APN can improve IR and atherosclerosis in mice; in a clinical trial study, APN was found to have anti-diabetic, anti-atherogenic, and inflammatory potential, proposing that lipocalin levels predicted the development of T2DM and coronary artery disease. Furthermore, lipocalin regulates glucose utilization and fatty acid oxidation, as well as the inflammatory response and anti-atherosclerotic processes [15]. Shaver et al. discovered that serum APN levels in DCM patients were lower than in diabetic patients alone and significantly lower than in normal controls, implying that clinical APN levels can be used to monitor early cardiomyopathy in diabetic patients [16]. A decrease in serum APN levels was also observed in the DCM rat model and was negatively correlated with the IR index, implying that APN may be involved in the development and progression of DCM via IR. Serum APN levels have been found to be highly correlated with other biochemical indicators in clinical studies. Serum APN levels, for example, were negatively correlated with FPG, triglyceride, total cholesterol, fasting insulin levels, and the homeostasis model assessment (HOMA)-IR index in the T2DM group. Myocardial adipoR1 protein expression was found to be positively related to APN levels and negatively related to fasting insulin (FINS) and HOMA-IR. Low APN levels were associated with metabolic disturbances in blood glucose and lipid levels, as well as IR. Furthermore, low levels of myocardial Adipo1R mRNA and protein expression were linked to decreased insulin sensitivity [17]. Leffler et al. proposed a key role of reduced serum APN concentrations and cardiac APN-Cx43 signaling dysfunction in this gender/E2-specific clinical problem in a study of female diabetic patients, linking ovarian hormone/E2 to worsening myocardial dysfunction in female diabetic patients for the first time [18].

Myocardial hypertrophy is one of the independent risk factors that contribute to a significantly higher incidence of cardiovascular disease and mortality, and it also plays a role in the pathogenesis of DCM. According to epidemiological studies, serum APN levels are inversely related to left ventricular hypertrophy [19]. APN^−/−^ mice were found to exacerbate myocardial hypertrophy caused by a high-fat diet (HFD) in adult C57 WT and APN^−/−^ mice fed with HFD [20]. Cardiac remodeling is severe in APN^−/−^ mice compared to wild-type mice, whereas cardiomyocyte hypertrophy and myocardial fibrosis are both reduced in wild-type mice after APN adenoviral overexpression [21]. APN protects the cardiomyocyte primarily by inhibiting extracellular signal-related kinases, activating adenosine monophosphate-activated protein kinase signaling in the myocardium, inhibiting cardiomyocyte hypertrophy or activating macrophage autophagy, and reducing cellular inflammation in the DCM rat model [22]. Meng et al. found that APN-modified bone marrow mesenchymal stem cells (BMSCs) could reduce TGF-β1/smad expression and thus suppress myocardial fibrosis in diabetic rats and HG-induced H9C2 cells [23]. Furthermore, by simultaneously activating nuclear factor erythroid 2-related factor 2 (NRF2) and Brahma-related gene 1 (BRG1), APN promotes HO-1 induction, reducing cardiac oxidative stress, improving cardiomyocyte hypertrophy, and preventing left ventricular dysfunction in diabetic patients [24]. In a diabetic rat model of myocardial ischemia–reperfusion injury, plasma APN and cardiac p-Akt expression were decreased, and the myocardium showed severe myocardial infarction and oxidative stress injury. In non-diabetic rats, postischemic treatment and increased plasma APN levels and cardiac p-Akt expression reduced myocardial injury [25]. Thus, it is clear that APN plays an important role in DCM, primarily through changes in protein expression or by mediating the corresponding regulation of signaling.

The evidence presented above suggests that low lipocalin levels in diabetic patients predispose them to the development of DCM. Could the increase in the expression of APN in the treatment of DCM still be unknown? APN supplementation may activate the adenylate-activated protein kinase signaling pathway and prevent the development of myocardial hypertrophy [22]. Exenatide treatment increased APN and HMW-APN (High molecular weight adiponectin) and the APPL1-AMPK-PPAR axis, decreased NF-κB and apoptosis, and improved cardiac function in diabetic rats, and these effects were controlled independently of glucose. These effects inversely confirmed that elevated APN levels contribute to protection against DCM [26].

#### Irisin

IR is a major contributor to the development of metabolic syndrome and T2DM, as well as DCM. Researchers have identified another class of brown fat and a new hormone that converts fat types, according to an article published in the journal Nature in January 2012, giving new hope to of clinical treatment of obesity [27]. Irisin is novel actin that is primarily expressed in the heart, skeletal muscle, liver, and kidney and is cleaved by its precursor fibronectin type III structural domain protein 5 (FNDC5). It has received considerable attention as an important cardioprotective factor and glucose metabolism regulator. Irisin is involved in many aspects of cardiovascular physiology and pathophysiology, and its expression is higher in cardiac muscle tissue than in skeletal muscle [27].

Irisin has previously been linked to increased insulin sensitivity, which has been linked to a reduction in DCM. Following palmitic acid (PA) treatment of H9C2 cells in rat cardiomyocytes to induce IR, irisin alleviated IR in H9C2 cells by inhibiting autophagy via the PI3K/Akt pathway, thereby providing cardiovascular protection [28]. It has also been demonstrated that irisin can reduce 78-kDa glucose-regulated protein (GRP78) and p-PERK/PERK protein expression levels, inhibits HG-induced oxidative stress in the endoplasmic reticulum, reduces myocardial hypertrophy, and thus have a cardioprotective effect [29]. Furthermore, through the mammalian target of the AMPK/rapamycin signaling pathway, irisin may play an important role in anti-apoptotic, anti-inflammatory, and anti-oxidative stress in HG-induced DCM [30]. Liu et al. reported that recombinant r-irisin (0.5 or 1.5 g/g body weight/day, I.P.) treatment did not alter blood glucose levels in diabetic mice, but inhibited HG-induced endothelial-mesenchymal transition (EndMT), thereby reducing cardiac fibrosis and left ventricular dysfunction in diabetic mice, whereas high-dose irisin failed to reduce myocardial fibrosis and ventricular dysfunction [31]. Lin et al. reduced DCM in type 2 diabetic mouse model by activating integrin αVβ5/AKT signaling and reducing oxidative & nitrosative stress in db/db mice and inhibited cardiomyocyte apoptosis, myocardial fibrosis, and cardiac hypertrophy in db/db mice by exogenous irisin supplementation, thereby reducing diastolic dysfunction and cardiac [32]. Thus, irisin plays an important role in DCM and has the potential to be used as an early clinical marker.

#### IGFBP7 (Insulin-like growth factor-binding protein 7)

IGFBP7 is an insulin-like growth factor regulator that binds competitively to insulin, preventing insulin from binding to the insulin receptor and causing IR. Furthermore, IGFBP7 regulates apoptosis, proliferation, and fibrosis in an autocrine and paracrine fashion. In a study of 271 acute dyspnoeic patients with and without acute HF, Kalayci et al. demonstrated the potential of IGFBP7 in improving the diagnosis of acute HF [33]. Serum IGFBP7 levels are highly correlated with echocardiographic abnormalities in cardiac hypertrophy, myocyte fibrosis, and diastolic dysfunction associated with vascular remodeling [16, 34]. Gandhi et al. discovered that patients with elevated baseline IGFBP7 concentrations were more likely to have an abnormal diastolic function in a 10-month follow-up of 124 patients with reduced ejection fraction heart failure, indicating the early predictive value of IGFBP7 in DCM [35].

### Immunoinflammatory response-related factors and oxidative stress-related indicators

#### TGF-β (Transforming growth factor-β)

TGF-β is a key mediator of inflammatory and immune responses, as well as a regulator of many biological processes including cell proliferation, differentiation, and apoptosis. TGF-β primarily activates the classical Smad signaling pathway, stimulates increased expression of extracellular matrix proteins, and inhibits the expression of extracellular matrix protein degrading enzymes, resulting in myocardial fibrosis and hypertrophy. Shaver et al. showed that plasma TGF-β levels in DCM patients were higher than in diabetic patients, and TGF-β plays a role in DCM [16]. Tan et al. discovered that TGF inhibitors improved ventricular diastolic disturbances in a T2DM mouse model [36]. Melatonin inhibited TGF-β/Smads signaling and LncRNA-MALAT1/miR-141-mediated NOD-like receptor thermal protein domain associated protein 3 (NLRP3) inflammasome activation, downregulated TGF-β, p-Smad2, p-Smad3, NLRP3, ASC expression, and reduced collagen to produce antifibrotic effects, ultimately improving cardiac dysfunction, according to Che et al. [37]. Furthermore, follicle inhibitors reduce the cardioprotective effects of DCM by inhibiting the TGF-β1-Smad3 pathway's antifibrotic effects. TGF-β has been shown in more animal models [38–42] to reduce myocardial fibrosis by inhibiting collagen production and deposition. Inhibiting the TGF-β/Smads signaling pathway can significantly reduce cardiac insufficiency in DM rats, thereby alleviating DCM. As a result, TGF-β is expected to be an important marker for DCM diagnosis and treatment.

#### Activin A

Activin A is a member of the TGF-β family and is found in a variety of tissues. Inflammation, tissue remodeling, and cytoprotection are all linked to activin A. Blumensatt et al. demonstrated that activin A released from T2DM patients’ epicardial adipose tissue inhibited insulin-induced phosphorylation of protein kinase B and its substrate by upregulating miR-143 levels, elucidating the role of activin A in metabolic disorders [43]. Activin A produced by epicardial adipose tissue (EAT) T2D inhibits insulin action by inducing miR-143 in cardiomyocytes via the insulin regulator oxysterol-binding protein-related protein 8 (ORP8) to inhibit the Akt pathway, thereby slowing the progression of cardiac insufficiency [43]. Activin A levels are strongly correlated with impaired myocardial glucose metabolism and high left ventricular mass/volume (LVMV) ratios in patients with T2DM early in the development of DCM; thus, increasing and maintaining high levels of activin A is a potentially deleterious effect on cardiomyocytes. Metformin treatment lowers activin A levels, and there is a critical and significant link between changes in plasma activin A levels and changes in LVMV ratios [44]. As a result, elevated activin A levels are more likely to result in DCM.

#### NLRP3 inflammatory

The expression of NLRP3 is increased in the hearts of diabetic mice. It has been shown that NLRP3 inflammatory vesicles are widely expressed in cardiac myocytes and that NLRP3 inflammasomes are activated in DCM. All of these findings support the hypothesis that NLRP3 inflammasomes are involved in the pathogenesis of DCM [45]. Metformin delays the onset of DCM in streptozocin (STZ)-induced C57BL/6 mice and neonatal mouse cardiomyocytes treated with HG. This effect is achieved by inhibiting the mTOR pathway and reducing cellular scorching in DCM [46]. Sirtuin 3 (SIRT3) deficiency exacerbated hyperglycemia-induced mitochondrial damage, increased ROS accumulation, promoted necroptosis, activated the NLRP3 inflammasome, and ultimately worsened DCM in mice, as shown by Song et al. in an STZ-induced diabetic mouse model [47]. Myocardial injury is induced in a cellular model of diabetes when HG stimulation activates the NLRP3 inflammasome and promotes IL-1β secretion in H9C2 cells and neonatal rat ventricular myocytes (NRVM) [48]. Does inhibition of NLRP3 activation improve DCM? Tilianin and syringin were used together by Yao et al. to improve cardiac function in diabetic rats by lowering levels of diabetes/hyperglycemia-induced oxidative stress and regulating the TLR4/NF-κB/NLRP3 and PGC1/SIRT3/mitochondrial pathways [49]. Folic acid slows the development of DCM by blocking NLRP3-mediated inflammatory vesicle-mediated cellular scorching through downregulation of the Hippo signaling pathway [50]. Inhibiting TGF-β1/Smad signaling and lowering NLRP3 inflammasome activation, the extract of Coriolus Versicolor protects against DCM in rats by decreasing myocardial fibrosis and inflammation [51]. Cardiovascular inflammation and fibrosis were reduced in NLRP3^−/−^ mice, and significant improvement in cardiac function. Meanwhile, inhibiting NLRP3 expression reduces caspase-1 and IL-1 protein expression under HG conditions, effectively reducing cell mortality in H9C2 cells [52]. Similarly, rosuvastatin was found to reduce cardiac dysfunction in DCM rats by inhibiting NLRP3 inflammasomes [53]. All of the above shreds of evidence suggest that targeting the NLRP3 inflammasome pathway may be an appropriate strategy for reducing DCM and cardiovascular disease in diabetic patients.

#### GDF-15 (growth differentiation factor-15)

Macrophages and cardiomyocytes produce GDF-15 as a response to oxidative stress and inflammation within the cell. Some diseases, such as cancer, heart disease, and heart failure, have been linked to increased GDF-15 levels [54]. According to Dominguez-Rodriguez et al., GDF-15 serum levels were significantly higher in patients with DCM compared to those without cardiomyopathy [55]. Changes in cardiac function (mitral isovolumic systolic time, systolic time, and isovolumic diastolic time values, all of which reflect diastolic function, were statistically significant compared with controls) as reflected by tissue doppler imaging were correlated with GDF-15 in a follow-up study of cardiomyopathy in patients with type 1 diabetes [56]. In conclusion, GDF-15 can be a reliable indicator for screening diabetic patients for DCM.

#### GRK2 (G protein-coupled receptor kinase 2)

A class of kinases known as G protein-coupled receptor kinases is responsible for the rapid desensitization of G protein-coupled receptors (GPCRs). The rapid attenuation of transduction signals in response to agonist stimulation seen in many GPCRs, such as opioid receptors, thromboxane receptors, 5-hydroxytryptamine receptors, and adrenergic receptors, is primarily caused by GRKs. Since the levels of GRK2 in the myocardium are similar to those found in the mononuclear cells of the peripheral blood, this suggests that GRK2 is an important regulator of the function of the heart.

The left ventricular wall thickness and systolic function of a DCM mouse model showed that GRK2 mRNA levels and expression were significantly higher in 12-week-old diabetic mice than in 8-week-old diabetic mice, as were collagen volume fraction (CVF), collagen-3 expression, P53 expression, and apoptosis in the myocardium. In the earliest stages of DCM, GRK2 expression is elevated in monocytes and myocardial tissue [57]. Clinically approved for the treatment of DCM, the GRK2 inhibitor paroxetine improves cardiac function in mouse models of the disease by restoring circulating Treg cell populations via targeting of the GRK2-PI3K-Akt pathway [58]. Treatment of diabetic mice with a GRK2 inhibitor for 2 weeks improved skeletal muscle glucose uptake and insulin signaling, as well as glucose tolerance (IGTT) and insulin sensitivity (ITT), confirming the effect of GRK2 inhibition on glucose extraction and insulin signaling. Attenuation of the inflammatory response and cytokine production, reduction of oxidative stress, and correction of the fetal gene expression pattern characteristic of DCM were all results of GRK2 inhibition in cardiac tissue [59].

Treatment with the β-blocker carvedilol or GRK2 inhibitors attenuates insulin-induced phosphorylation of extracellular regulated protein kinases and phosphodiesterase 4D (PDE4D) induction in 2-adrenergic receptor (β2AR) or β-arrestin2 knockout mice fed with HFD, thereby reducing systolic dysfunction in DCM [60]. Increased levels of macrophage migration inhibitory factor (MIF) are associated with hyperglycemia and have been linked to the pathogenesis of cardiomyopathy in people with T2DM. Clinical research revealed that both diabetic patients and those with left ventricular diastolic dysfunction had significantly increased plasma MIF levels. The expression of MIF and GRK2 was significantly upregulated in H9C2 cardiomyocytes as a function of glucose concentration, and GRK2-mediated increases in MIF levels were linked to cardiac insufficiency in diabetic patients [61].

In summary, given GRK2’s proven involvement in both IR and systolic heart failure, it's clear that this protein is worth further investigation as a therapeutic target. Because of its role as a clinical biomarker for early DCM, GRK2 has the potential to be a game-changing application in the treatment of diabetes and its cardiovascular complications.

### Factors associated with myocardial injury and remodeling.

#### Apelin

Canadian researchers reported the presence of an angiotensin receptor AT1-related receptor protein in 1993; in 1998, Japanese researchers identified and isolated the natural ligand of APJ from the bovine stomach; they named this ligand apelin [62]. The normal level of apelin in vivo is between 3–4 ng/mL, and it can form heterodimers with other cellular receptors to help it achieve its richer biological functions [63]. Apelin/APJ is highly conserved across species and has been shown to have crucial roles in the cardiovascular system. Through mechanisms including regulation of hormone secretion (for example, by interfering with insulin synthesis), Apelin plays a role in controlling the progression of DCM. The amino acid apelin raises intracellular Ca^2+^ concentration, which in turn boosts cardiac output, in heart failure patients [64], and has a remarkable inotropic effect that is beneficial to the heart [65–67]. In both humans and animals, researchers have found inverse relationships between plasma apelin levels and LV mass index and between apelin and APJ myocardial expression. In conditions of pressure overload or DCM, where muscle force and cardiac output would otherwise decline, an increase in plasma apelin levels may serve as a compensatory mechanism [68]. Furthermore, apelin suppresses cell growth and migration by dephosphorylating specific extracellular signaling kinases and blocking amidated cyclase. Effects of vasodilation and hypotension caused by increased exogenous apelin are achieved by increasing NO production [69]. In addition, apelin is also involved in the angiogenic process.

Endothelial cell dysfunction and microcirculatory damage contribute to the development of DCM. Apelin increases insulin-stimulated vasodilation and decreases AT-II-induced vasoconstriction in subjects with central obesity in an endothelium-dependent and non-independent fashion [70]. In endothelial cell-specific APJ^−/−^ mice and endothelial cell studies, Li et al. reported that apelin improved endothelial cell dysfunction to alleviate diabetic cardiopathy by improving the NFB pathway dependent on APJ activation, reducing the expression of apoptotic and adhesion molecules, and increasing proliferation, angiogenesis, and the expression of E-cadherin, VEGFR 2, and Tie-2 [71]. A paracrine role for the endothelial sirt3-apelin axis in the regulation of glucose transport proteins in cardiomyocytes was suggested by the observation by Zeng et al. that apelin-induced glucose uptake was diminished in endothelial cells of sirt3-deficient mice [72]. Apelin treatment effectively decreased HFD-induced cardiac hypertrophy and systolic dysfunction in Apelin^−/−^ mice and alleviated the ensuing systolic dysfunction [73].

Apelin’s biological effects are bolstered by its ability to indirectly influence the expression of other proteins, which has both direct and additional protective effects. SIRT3, a mitochondria-localized protein with deacetylase activity, is involved in hypertrophy through the Apelin/Sirt3 signaling pathway. To prevent cardiac hypertrophy from occurring during development, inhibiting the Apelin/Sirt3 signaling pathway reduces ROS-producing enzymes [74]. Epimedoside suppressed DCM progression in db/db mice by enhancing Apelin/Sirt3-mediated mitochondrial function [75]. Increased vessel density and decreased DCM can be achieved through overexpression of apelin due to its activation of Sirt3 and upregulation of vascular endothelial growth factor/VEGF receptor 2 (VEGFR2) and angiogenesis/tissue interconnectivity factor 2 (Ang-1/Tie-2) expression [72]. Increased cardiac sirt3 expression and decreased ROS formation have been shown in multiple studies to be effective treatments for DCM [72, 76]. Sirt3 is also necessary for the angiogenesis and autophagy of the heart that is induced by apelin in diabetics [77, 78]. The PAI-1 inhibitor apelin prevents AT-II-induced fibrosis in mice by activating the TGF-β pathway [79]. Obese mice treated with Fc-apelin for an extended period of time have improved cardiac output and lessened cardiac fibrosis [80]. In rats with myocardial infarction-induced heart failure, apelin inhibits fibrosis and PI3K/Akt activity via the TGF-β1/smad 2 pathway to reduce oxidative stress [81, 82]. It is therefore evident that apelin has cardioprotective effects via activating sirt3 and other mechanisms.

#### Heart-type fatty acid binding protein (hFABP)

High cardiac specificity and high expression levels characterize a novel small cytoplasmic protein called heart-type fatty acid binding protein (hFABP). The molecular weight of the cytoplasmic protein that binds fatty acids in the heart is 15 kDa, and it contains 132 amino acids. It gets fat molecules from the bloodstream to the mitochondria. The main biological effect of FABP is to participate in fatty acid metabolism, which was discovered by R.K. Okner et al. in 1972. FABP is primarily expressed in cardiac muscle, skeletal muscle, small intestine, kidney, liver, and other tissues. The hFABP has been linked to a number of health problems, including heart attacks, cardiac abnormalities, strokes, and sleep apnea. One of the earliest diagnostic markers of acute coronary syndrome (ACS) is an elevation of hFABP in the blood after myocardial ischemic injury; this elevation occurs as early as 1–3 h after the onset of chest pain, peaks at 6–8 h, and plasma levels return to normal within 24–30 h [83]. Significantly increased hFABP levels were observed in type 2 diabetic patients with new-onset myocardial injury, as determined by the work of Akbal et al. [84]. Shearer et al. show that hFABP is linked to IR and glucose uptake in diabetic mice [85]. Patients with spinal cord injuries have higher serum levels of the hFABP, and this is strongly correlated with carotid intima-media thickness. Patients with impaired fasting glucose (IFG) and impaired glucose tolerance (IGT) may find serum hFABP levels to be an informative indicator of myocardial function [86, 87]. An additional clinical study involving 50 prediabetic patients confirmed the utility of hFABP serum levels in prodromal diabetes and their close relationship with IR. IR is the most important factor in explaining why prediabetic patients have a higher risk of cardiovascular disease compared to normoglycemic patients. Serum hFABP levels are an early indicator of subclinical cardiovascular disease, and they are increased in patients with pre-diabetes (CVD) [86].

Increased oxidative stress is a major contributor to the development of chronic inflammation and a major cause of DCM induction. Evidence suggests that Anchomanes difformis may have antioxidant and anti-inflammatory effects. By decreasing levels of hFABP in the hearts of diabetic-treated animals, Anchomanes difformis reduces pro-inflammatory cytokines, increases anti-inflammatory markers, enhances antioxidant defense, and mitigates the progression of DCM [88]. To ameliorate streptozotocin-induced cardiomyopathy in diabetic rats, an aqueous extract of the leaves of Fructus Lindl was found to reduce hyperlipidemia as well as oxidative stress. This was accomplished by lowering the expression of urotensin II and FABP3 [89]. Researchers investigating the role of lipotoxicity in DCM found that elevated FABP3 expression in the diabetic heart combined with elevated expression of other lipid metabolites causes mitochondrial damage, phosphorylation uncoupling, and ATP deficiency, ultimately leading to diabetic cardiac insufficiency [90]. Akbal et al. found that elevated FABP3 expression in the diabetic heart contributed to mitochondrial damage, phosphorylation uncoupling, and ATP deficiency, all of which contributed to diabetic cardiac insufficiency [84]. Therefore, hFABP is preferable as a marker of myocardial injury. These results provide further evidence that this factor may aid in the detection of DCM at an earlier stage.

#### Galactin-3 (Gal-3)

Cardiac fibrosis and inflammation are both strongly linked to Gal-3, the only chimeric galactose lectin family protein in spinal animals [91]. By directly or indirectly activating fibroblasts and promoting the expression of other related factors that affect collagen synthesis in fibroblasts, Gal-3 promotes myocardial fibrosis by interacting with the renin–angiotensin–aldosterone system and mediated by TGF-β and Hippo pathways [92, 93]. Cardiac fibrosis plays a key role in the development of DCM, Gal-3 can be involved in the progression of DCM through a number of different mechanisms. Hyperglycemia leads to the accumulation of advanced glycation end products (AGEs), oxidative stress products, and pro-apoptotic pathways [94]. Gal-3 promotes oxidative stress and mediates the pro-fibrotic effects of (angiotensin II) AT-II and aldosterone [95, 96]. Chronic heart failure is a common manifestation of DCM, and sodium-glucose co-transporter protein 2 inhibitor (SGLT2i) improves outcomes in patients with diabetes and HF. After 3 years of follow-up, T2DM patients who were treated with SLGT2i were very likely to develop HFpEF if their Gal-3 levels were higher than 10 ng/ml. In recent clinical trials, Gal-3 levels were a good predictor of HFpEF [97]. Another clinical study found that 155 HF patients with a reduced ejection fraction had low-dose dobutamine echocardiography and blood sampling for biomarker measurements. Increased levels of Galectin-3 were observed in those with impaired glucose metabolism and HF. However, there was no independent effect on plasma Gal-3 levels due to reduced LV systolic reserve in patients with DM HF (on the other hand, in patients with HF, plasma levels of Gal-3 were higher in patients with impaired glucose metabolism) [98]. An increased level of Gal-3 is associated with reduced global longitudinal strain in animal models of HF, and this association is strengthened in diabetic patients with a mildly low ejection fraction. Gal-3 may be useful for diagnosing left ventricular dysfunction and DCM at an early stage [99]. Predicting long-term cardiovascular mortality in patients with systolic HF and contributing to risk stratification in patients with HF, Gal-3 levels were measured using the VIDAS^®^ assay [100]. Based on this evidence, the Food and Drug Administration (FDA) has authorized the use of Gal-3 as a novel biomarker for predicting adverse cardiovascular events, such as HF [101]. Furthermore, Gal-3 was associated with left ventricular diastolic dysfunction and elevated pulmonary artery systolic pressure in young, obese patients with no history of cardiovascular disease, suggesting a role in preclinical screening for metabolic heart disease [102]. Recently, Flores-Ramirez et al. analyzed Gal-3’s potential impact on DCM diagnosis [99]. A simple and reproducible echocardiographic tool for the assessment and follow-up of DCM, reduced overall longitudinal strain, is shown in this study to be associated with elevated Gal-3 levels in diabetic patients with mildly reduced ejection fraction [103]. Gal-3 levels were linked to higher body mass index (BMI), waist circumference, obesity, and triglycerides in a cross-sectional study of 2946 patients [104]. Decreased Gal-3 levels are beneficial for DCM. Glibenclamide has been shown to prevent and alleviate DM atrial structural remodeling by activating the NLRP3-inflammasome/caspase-1/Gal-3 signaling pathway, thereby decreasing NLRP3 and Gal-3 expression that is induced by DM [105].

A clinical trial conducted by Jelena et al. on 189 patients with T2DM and/or arterial HT reported a positive correlation between Gal-3 levels and T2DM and HT, as well as a positive correlation with left ventricular weight, suggesting a potential role of Gal-3 for the early detection of structural and functional changes in the myocardium [106]. Myocardial inflammation and fibrosis are accompanied by changes in Gal-3 concentrations [107]. Further studies have confirmed its mechanism of inducing cardiomyocyte changes, including hypertrophy, increased fibrosis, stiffness, and impaired relaxation triggered by a pro-inflammatory environment through adipose tissue dysfunction, which can use Gal-3 as a biomarker of myocardial functional changes in high calorie-induced fibrosis [108]. Reducing ventricular dilation, congestion, and fibrosis through the suppression of Gal-3 mRNA expression in Mst1-TG mice, which model DCM with high levels of cardiac Gal-3, is a promising therapeutic approach [109]. Silencing Gal-3 reduces myocardial dysfunction in DCM animals by downregulation of macrophage infiltration and inflammatory cytokine release triggered by HG via the Gal-3/NF-κB p65 regulatory network in STZ-induced DCM in mice [110]. Taking into account the foregoing, it seems reasonable to conclude that Gal-3 can be used as a prognostic marker for heart failure and is also well indicated in diabetic myocardium and that inhibition of Gal-3 to reduce inflammation and fibrosis is a potential therapeutic approach to prevent heart disease.

#### Cardiotrophin-1 (CT-1)

CT-1 was found for the first in mouse cardiac embryonic stem cells in 1995 by Pennica et al. [111]. CT-1 is mainly produced by cardiac myocytes and non-cardiac myocytes in the heart and is secreted into the peripheral circulation via the coronary sinus [112]. CT-1, a member of the glycoprotein 130 families, is a powerful inducer of cardiomyocyte enlargement in vitro and is consequently also known as a cardiotrophin [113]. Although CT-1 stimulates cardiomyocyte hypertrophy, it can also exert cardioprotective effects by activating three distinct signaling pathways: (1) the Janus kinase/signal transducer and activator of transcription (JAK/STAT) pathway, (2) the extracellular receptor kinase-1/2 (ERK1/2) pathway, and (3) the phosphatidylinositol 3-OH kinase (PI3K)/Akt pathway [114]. Studies reveal that hereditary hypertension, coronary artery disease, myocardial infarction, dilated cardiomyopathy, and heart failure all have higher CT-1 expression, suggesting a link between CT-1 and the development of these conditions [115, 116]. It has been found that CT-1 correlates with LV mass index and severity of LV failure, suggesting that it plays a significant role in dilated cardiomyopathy through its involvement in myocardial remodeling [115]. Furthermore, Monserrat et al.[117] discovered that CT-1 was connected to significantly greater levels of CT-1 expression in hypertrophic cardiomyopathy and was directly related to the degree of LVH. Cardiotrophin-1 increased sarcoplasmic reticulum calcium leak and arrhythmogenesis in adult rat ventricular myocytes, as shown by research conducted by Ruiz-Hurtado et al. [118]. Inhibition of JAK2, myosin light chain kinase, and cardiac fibroblast signaling pathways by CT-1 results in changes in intracellular Ca^2+^ concentrations and cell membrane protein alterations, which in turn affect ventricular remodeling (as demonstrated by Freed et al. [119]). Controlling the onset and progression of diabetic cardiomyopathy, CT-1 regulates glucose and lipid metabolism, corrects IR, and is implicated in the development of glucose- and insulin-induced cardiac hypertrophy [120]. Results from the study by Liu et al. showed that in neonatal rat cardiomyocytes, pioglitazone prevented the increase in CT-l expression in high-glucose, high-insulin-induced hypertrophic cardiomyocytes, indicating that CT-1 is a major regulator of fat and glucose metabolism with potential applications in the treatment of obesity and IR [121]. 3D speckle-tracking echocardiography and tissue Doppler imaging were used in a clinical trial by Sonia et al. [122], who compared 55 children with type 1 diabetes to 55 healthy controls and found that diabetic patients exhibited right ventricular and left ventricular systolic-diastolic dysfunction compared to controls. The best predictor of left ventricular systolic dysfunction was CT-1 (sensitivity: 69%).In an observational study of 93 patients with T2DM, Gamella-Pozuelo [123] discovered that diabetic patients had significantly higher plasma CT-1 levels and were more likely to have ventricular hypertrophy. Tsutamoto et al. [124] discovered increased plasma CT-1 levels in DCM patients, as well as a substantial positive connection with the left ventricular mass index. Furthermore, Briana et al. discovered higher levels of CT-1 in cord blood samples from term singleton pregnant women of greater than gestational age, implying gigantic fetuses and cardiomyocyte/diastolic dysfunction [125]. Clinical evidence suggests that CT-1 is also involved in the structural remodeling of the ventricles in DCM patients. CT-1 may perform a protective role in diabetic mice by enhancing -cell function and survival, in addition to its influence on cardiac remodeling [126]. CT-1 can also stimulate the secretion of atrial natriuretic peptide and B-type natriuretic peptide by acting as a G protein-coupled receptor stimulator [127]. In conclusion, higher CT-1 levels in diabetic patients may promote cardiomyocyte hypertrophy and increased myocardial collagen content, and detecting changes in plasma CT-1 levels in diabetic patients may aid in the early diagnosis and prognosis of DCM.

### Current clinical biomarkers used to detect DCM

In addition to the aforementioned biomarkers, several others have been used in clinical practice that is neither particularly specific nor sensitive but are still indicative (Table 1). Myocardial injury can be detected at an early stage by measuring the levels of troponin T (cTnT), troponin I (cTnI), and creatinine kinase-MB (CK-MB) in the clinic. CTn is a sensitive and highly specific marker of myocardial damage for the diagnosis of cardiac disorders due to its distinctive amino acid sequence in the clinic. It is also commonly utilized clinically for the early detection of the acute coronary syndrome and is a good biochemical biomarker for the diagnosis of myocardial damage and myocardial infarction (ACS). There is currently insufficient evidence supporting cTnI as a biological diagnostic of DCM, and it is mostly utilized as an auxiliary signal, despite the fact that cTnI levels in the blood of DCM patients are higher than in the diabetic group. Elevated levels of ANP and BNP are a strong indicator of heart failure in patients with myocardial hypertrophy. Increased inflammation is associated with an increased risk of DCM. The serum C-reactive protein (CRP) is a sensitive predictor of cardiovascular events and is critical for predicting cardiovascular events and determining treatment prognosis [128]. For several cardiac conditions, including congestive heart failure and acute myocardial infarction, the level of ANP and BNP reference index is crucial. Plasma ANP concentration in DC patients can serve as a reference marker for identifying early DCM, and ANP levels can represent the presence and degree of early cardiac dysfunction, i.e., asymptomatic left ventricular dysfunction. Diastolic insufficiency is a reference criterion for early diabetic cardiomyopathy and is associated with significantly higher blood BNP levels in type 2 diabetic patients. Predicting the occurrence of cardiovascular events and estimating the prognosis of treatment are both crucial applications of serum CRP, which is a more sensitive predictor of cardiovascular events. Increased CRP levels are a sensitive serological test for the diagnosis of DCM because they represent cardiac injury to some extent and indicate the likelihood of co-infection in patients with DCM compared to those with diabetes alone. C-reactive protein levels are more than three times higher in patients with diabetes and coronary artery disease than in those with diabetes alone, reflecting some extent of myocardial damage and predicting the tendency to complicate infection. This is true even in the absence of clinical symptoms and cardiac dysfunction. Given its central role in regulating angiogenesis, Ang II is a promising therapeutic target for the prevention and treatment of DCM [129]. Hyperplastic hypertrophy of cardiomyocytes and interstitial collagen deposition are reflected in increased Ang II levels in cardiac tissue [130]. Since myocardial fibrosis is widespread in DCM and a rise in CTGF levels often before clinical indications of cardiac fibrosis, the CTGF test is relevant in evaluating myocardial fibrosis and its prognosis [131]. Individually, these markers aid in the prediction of diabetes and cardiovascular disease, but taken as a whole, they are insufficient. Still, they aren’t adequate as a DCM early warning system. Therefore, it is essential to discover new biomarkers that can be used to predict disease onset and progression.

**Table 1:** Summary table of common clinical test markers

Name	Major mechanism	Change	References
ANP,BNP	1) Affecting blood pressure through acting on vascular smooth muscle cells 2) Preventing vascular smooth muscle cell growth and proliferation and vascular fibrosis 3) Acting on the heart itself to suppress cardiac hypertrophy and fibrosis	Up	[132, 133]
Ang II	1) Cardiac hypertension and oxidative stress 2) Promoting and suppressing coronary angiogenesis, via VEGF and TSP-1 produced from hypertrophied cardiomyocytes under chronic Hypoxia	Up	[134, 135]
C peptide	Na^+^, K^+^-ATPase and PKC-α	Down	[136]
cTn	Oxidative stress and inflammatory damage	Up	[137] [138] [139]
CK-MB	Apoptosis, Myocardial injury, Oxidative Stress and Inflammation	Up	[57, 140]
CTGF	Cardiac fibrosis,	Up	[131]
hs-CRP	Inflammation, oxidative stress	Up	[128]
Hcy	Oxidative stress	Up	[141]
IL-1β	Promoting cardiomyocyte apoptosis by activating the IRAK-2/CHOP pathway of endoplasmic reticulum stress	Up	[142]
TNF-α	Cardiac fibrosis, inflammation	Up	[143, 144]
IL-6	Cardiac remodeling, fibrosis and cardiomyocyte apoptosis	Up	[144]
8-OHdG	Oxidative stress and DNA oxidation	Up	[145]
MMPs	Cardiac fibrosis	Down	[146, 147]

βARK1, β-adrenergic receptor kinase 1; CTGF,connective tissue growth factor; cTn,cardiac troponin; CK-MB, creatine kinase MB isoenzyme; Hcy, homocysteine; hs-CRP, hypersensitive-c-reactive-protein; 8-OHdG, 8-hydroxy-2 deoxyguanosine; MMPs,matrix metalloproteinase. VEGF,vascular endothelial-derived growth factor;TSP-1, thrombospondin 1

## Conclusion and perspectives

Multiple factors, including but not limited to glucose levels, hypertension, hyperlipidemia, and oxidative stress, have been related to DCM. DCM should be detected before significant heart damage occurs, making early and asymptomatic detection of DCM crucial. The clinical diagnosis of diabetic heart disease, especially in its early stages, still lacks clinical biomarkers with high specificity and sensitivity, despite major efforts to establish adequate diagnostic biomarkers for DCM. As more is learned about the causes of DCM, more and better clinical biomarkers are being identified (Table 2). Changes in plasma CT-1 levels and hFABP, for instance, may help in the early diagnosis and prognosis of DCM; GRK2 levels are generally altered early in DCM and can be used for early diabetic myocardial prediction. IGFBP-7 levels are elevated early in DCM and are the best predictor of right ventricular systolic dysfunction. The CTn level, which reflects the extent of myocardial injury, is a good predictor of early DCM, although it can’t confirm the diagnosis. The severity of the myocardial injury is reflected in the levels of apelin and hFABP, while Gal-3 and TGF-β may be used as a marker of cardiac fibrosis in the intermediate and late phases of DCM. HF is more likely to occur in patients with cardiac hypertrophy who have excessive levels of ANP and BNP, and this risk is further worsened in patients with DCM. In addition, the serum CRP is a reliable predictor of DCM outcome. To properly evaluate patients with DCM and provide an accurate diagnosis, it is not advised to rely on a single biomarker; rather, it is recommended that numerous biomarkers be used together. Finally, there is an urgent need for deeper study of the illness process and the hunt for more reliable biomarkers. In order to reduce the mortality, it is crucial that practitioners identify DCM at an early stage and begin treatment.

**Table 2:** Summary table of potential novel clinical biomarkers

Name	Major mechanism	Change	References
Irisin	1) Increasing insulin sensitivity 2) Inhibiting autophagy via the PI3K/Akt pathway 3) Inhibiting HG-induced oxidative stress via AMPK/rapamycin signaling pathway 4) Reducing oxidative & nitrosative stress via integrin αVβ5/AKT signaling	Up	[28–30, 32]
IGFBP7	Regulating apoptosis, proliferation, and fibrosis	Up	[33]
TGF-β	Inhibiting collagen production and deposition through Smad signaling pathway	Up	[38–42]
Activin A	Inhibiting insulin action through Akt pathway	Up	[43]
GRK2	improving cardiac function by restoring circulating Treg cell populations via targeting of the GRK2-PI3K-Akt pathway	Up	[57, 58]
hFABP	IR and glucose uptake	Up	[85]
CT-1	Cardiac fibrosis, ROS Cardiac hypertension	Up	[113] [148]
Gal-3	Promoting cardiac fibrosis and inflammation through TGF-β and Hippo pathways	Up	[92, 93]

CT-1, Cardiotrophin-1; Gal-3, galactin-3; GRK2, G protein-coupled receptor kinase 2**;** hFABP, heart-type fatty acid binding protein; HG, high glucose; IGFBP7, insulin-like growth factor-binding protein 7; TGF-β,transforming growth factor-β

## Data Availability

Not applicable.

## Abbreviations

ACS: Acute coronary syndrome
AGEs: Advanced glycation end products
AMPK: AMP-activated protein kinase
Ang-1/Tie-2: Angiogenesis/tissue interconnectivity factor 2
AKT: Adenylate kinase
APN: Adiponectin
ASC: Apoptosis-associated speck-like protein containing a caspase recruitment domain
AT-II: Angiotensin II
BMSCs: Bone marrow mesenchymal stem cells
CKMB: Creatinine kinase-MB
CRP: C-reactive protein
cTnI: Troponin I
cTnT: Troponin T
CVD: Cardiovascular disease
CVF: Collagen volume fraction
DCM: Diabetic cardiomyopathy
EAT-T2D: Epicardial adipose tissue from patients with type 2 diabetes
EndMT: Endothelial-mesenchymal transition
FNDC5: Fibronectin type III structural domain protein 5
FINS: Fasting insulin
FPG: Fasting plasma glucose
Gal-3: Galactin-3
GDF-15: Growth differentiation factor-15
GPCRs: G protein-coupled receptor kinase 2
GRK2: G protein-coupled receptor kinase 2
GRP78: Glucose-regulated protein 78
HF: Heart failure
hFABP: Heart-type fatty acid binding protein
HFD: High-fat diet
HG: High glucose
HOMA: Homeostasis model assessment
HMW-APN: High molecular weight adiponectin
HT: Hypertension
IDF: International diabetes federation
IGFBP7: Insulin-like growth factor-binding protein 7
IGTT: Glucose tolerance
IL-1: Interleukin 1
IR: Insulin resistance
ITT: Insulin sensitivity
LV: Left ventricular weight
LVMV: Left ventricular mass/volume
MIF: Migration inhibitory factor
miR-143: MicroRNA-143
NLRP3: NOD-like receptor thermal protein domain associated protein 3
NRF2: Nuclear factor erythroid 2-related factor 2
MAPK: Mitogen-activated protein kinase
MIF: Migration inhibitory factor
MMPs: Matrix metalloproteinases
ORP8: Oxysterol binding related proteins 8
PGC1: Peroxisome proliferator-activated receptor-γ coactivator 1
RAAS: Renin–angiotensin–aldosterone system
ROS: Reactive oxygen species
SGLT2i: Sodium glucose co-transporter protein 2 inhibit
T2DM: Type 2 diabetes mellit
TGF-β1: Transforming growth factor
TLR4: Toll-like receptor
VEGFR 2: Vascular endothelial growth factor receptor-2

## References

[1] IDF Diabetes Atlas 10th edition. 2021.

[2] Gulsin GS, Athithan L, McCann GP (2019). Diabetic cardiomyopathy: prevalence, determinants and potential treatments. Ther Adv Endocrinol Metab.

[3] Paulus WJ, Tschope C, Sanderson JE, Rusconi C, Flachskampf FA, Rademakers FE (2007). How to diagnose diastolic heart failure: a consensus statement on the diagnosis of heart failure with normal left ventricular ejection fraction by the Heart Failure and Echocardiography Associations of the European Society of Cardiology. Eur Heart J.

[4] Kannel WB, Hjortland M, Castelli WP (1974). Role of diabetes in congestive heart failure: the Framingham study. Am J Cardiol.

[5] Thrainsdottir IS, Aspelund T, Thorgeirsson G, Gudnason V, Hardarson T, Malmberg K (2005). The association between glucose abnormalities and heart failure in the population-based Reykjavik study. Diabetes Care.

[6] Nichols GA, Hillier TA, Erbey JR, Brown JB (2001). Congestive heart failure in type 2 diabetes: prevalence, incidence, and risk factors. Diabetes Care.

[7] Bertoni AG, Hundley WG, Massing MW, Bonds DE, Burke GL, Goff DC (2004). Heart failure prevalence, incidence, and mortality in the elderly with diabetes. Diabetes Care.

[8] Miki T, Yuda S, Kouzu H, Miura T (2013). Diabetic cardiomyopathy: pathophysiology and clinical features. Heart Fail Rev.

[9] Boudina S, Abel ED (2007). Diabetic cardiomyopathy revisited. Circulation.

[10] Boonman-de Winter LJ, Rutten FH, Cramer MJ, Landman MJ, Liem AH, Rutten GE (2012). High prevalence of previously unknown heart failure and left ventricular dysfunction in patients with type 2 diabetes. Diabetologia.

[11] Nichols GA, Gullion CM, Koro CE, Ephross SA, Brown JB (2004). The incidence of congestive heart failure in type 2 diabetes: an update. Diabetes Care.

[12] Dei Cas A, Spigoni V, Ridolfi V, Metra M (2013). Diabetes and chronic heart failure: from diabetic cardiomyopathy to therapeutic approach. Endocr Metab Immune Disord Drug Targets.

[13] Devereux RB, Roman MJ, Paranicas M, O'Grady MJ, Lee ET, Welty TK (2000). Impact of diabetes on cardiac structure and function: the strong heart study. Circulation.

[14] Iribarren C, Karter AJ, Go AS, Ferrara A, Liu JY, Sidney S (2001). Glycemic control and heart failure among adult patients with diabetes. Circulation.

[15] Qi C, Mao X, Zhang Z, Wu H (2017). Classification and differential diagnosis of diabetic nephropathy. J Diabetes Res.

[16] Shaver A, Nichols A, Thompson E, Mallick A, Payne K, Jones C (2016). Role of serum biomarkers in early detection of diabetic cardiomyopathy in the West Virginian population. Int J Med Sci.

[17] Li J, Su S, Zong X (2014). Analysis of the association between adiponectin, adiponectin receptor 1 and diabetic cardiomyopathy. Exp Ther Med.

[18] Leffler KE, Abdel-Rahman AA (2019). Estrogen-dependent disruption of adiponectin-connexin43 signaling underlies exacerbated myocardial dysfunction in diabetic female rats. J Pharmacol Exp Ther.

[19] Mitsuhashi H, Yatsuya H, Tamakoshi K, Matsushita K, Otsuka R, Wada K (2007). Adiponectin level and left ventricular hypertrophy in Japanese men. Hypertension.

[20] Guo R, Zhang Y, Turdi S, Ren J (2013). Adiponectin knockout accentuates high fat diet-induced obesity and cardiac dysfunction: role of autophagy. Biochim Biophys Acta.

[21] Shibata R, Izumiya Y, Sato K, Papanicolaou K, Kihara S, Colucci WS (2007). Adiponectin protects against the development of systolic dysfunction following myocardial infarction. J Mol Cell Cardiol.

[22] Shibata R, Ouchi N, Ito M, Kihara S, Shiojima I, Pimentel DR (2004). Adiponectin-mediated modulation of hypertrophic signals in the heart. Nat Med.

[23] Meng K, Cai H, Cai S, Hong Y, Zhang X (2021). Adiponectin modified BMSCs alleviate heart fibrosis via inhibition TGF-beta1/Smad in diabetic rats. Front Cell Dev Biol.

[24] Li H, Yao W, Irwin MG, Wang T, Wang S, Zhang L (2015). Adiponectin ameliorates hyperglycemia-induced cardiac hypertrophy and dysfunction by concomitantly activating Nrf2 and Brg1. Free Radical Biol Med.

[25] Cao C, Liu HM, Li W, Wu Y, Leng Y, Xue R (2020). Role of adiponectin in diabetes myocardial ischemia-reperfusion injury and ischemic postconditioning. Acta Cir Bras.

[26] XiaoTian L, QiNan W, XiaGuang G, WuQuan D, Bing C, ZiWen L (2016). Exenatide activates the APPL1-AMPK-PPARalpha axis to prevent diabetic cardiomyocyte apoptosis. J Diabetes Res.

[27] Bostrom P, Wu J, Jedrychowski MP, Korde A, Ye L, Lo JC (2012). A PGC1-alpha-dependent myokine that drives brown-fat-like development of white fat and thermogenesis. Nature.

[28] Song R, Zhao X, Cao R, Liang Y, Zhang DQ, Wang R (2021). Irisin improves insulin resistance by inhibiting autophagy through the PI3K/Akt pathway in H9c2 cells. Gene.

[29] Li X, Zhang DQ, Wang X, Zhang Q, Qian L, Song R (2022). Irisin alleviates high glucose-induced hypertrophy in H9c2 cardiomyoblasts by inhibiting endoplasmic reticulum stress. Peptides.

[30] Deng J, Zhang N, Chen F, Yang C, Ning H, Xiao C (2020). Irisin ameliorates high glucose-induced cardiomyocytes injury via AMPK/mTOR signal pathway. Cell Biol Int.

[31] Liu X, Mujahid H, Rong B, Lu QH, Zhang W, Li P (2018). Irisin inhibits high glucose-induced endothelial-to-mesenchymal transition and exerts a dose-dependent bidirectional effect on diabetic cardiomyopathy. J Cell Mol Med.

[32] Lin C, Guo Y, Xia Y, Li C, Xu X, Qi T (2021). FNDC5/Irisin attenuates diabetic cardiomyopathy in a type 2 diabetes mouse model by activation of integrin alphaV/beta5-AKT signaling and reduction of oxidative/nitrosative stress. J Mol Cell Cardiol.

[33] Kalayci A, Peacock WF, Nagurney JT, Hollander JE, Levy PD, Singer AJ (2020). Echocardiographic assessment of insulin-like growth factor binding protein-7 and early identification of acute heart failure. ESC Heart Fail.

[34] Guo XH, Liu LX, Zhang HY, Zhang QQ, Li Y, Tian XX (2014). Insulin-like growth factor binding protein-related protein 1 contributes to hepatic fibrogenesis. J Dig Dis.

[35] Gandhi PU, Gaggin HK, Sheftel AD, Belcher AM, Weiner RB, Baggish AL (2014). Prognostic usefulness of insulin-like growth factor-binding protein 7 in heart failure with reduced ejection fraction: a novel biomarker of myocardial diastolic function?. Am J Cardiol.

[36] Tan SM, Zhang Y, Wang B, Tan CY, Zammit SC, Williams SJ (2012). FT23, an orally active antifibrotic compound, attenuates structural and functional abnormalities in an experimental model of diabetic cardiomyopathy. Clin Exp Pharmacol Physiol.

[37] Che H, Wang Y, Li H, Li Y, Sahil A, Lv J (2020). Melatonin alleviates cardiac fibrosis via inhibiting lncRNA MALAT1/miR-141-mediated NLRP3 inflammasome and TGF-beta1/Smads signaling in diabetic cardiomyopathy. FASEB J.

[38] Meng S, Yang F, Wang Y, Qin Y, Xian H, Che H (2019). Silymarin ameliorates diabetic cardiomyopathy via inhibiting TGF-beta1/Smad signaling. Cell Biol Int.

[39] Li R, Qi C, Feng Q, Ding P, Kang L, Chi J (2020). Alprostadil alleviates myocardial fibrosis in rats with diabetes mellitus through TGF-beta1/Smad signaling pathway. Minerva Endocrinol.

[40] Zhang Y, Cui L, Guan G, Wang J, Qiu C, Yang T (2018). Matrine suppresses cardiac fibrosis by inhibiting the TGFbeta/Smad pathway in experimental diabetic cardiomyopathy. Mol Med Rep.

[41] Zhang Y, Li Y, Huang X, Zhang F, Tang L, Xu S (2020). Systemic delivery of siRNA specific for silencing TLR4 gene expression reduces diabetic cardiomyopathy in a mouse model of streptozotocin-induced type 1 diabetes. Diabetes Ther.

[42] Li G, Yang L, Feng L, Yang J, Li Y, An J (2020). Syringaresinol protects against type 1 diabetic cardiomyopathy by alleviating inflammation responses, cardiac fibrosis, and oxidative stress. Mol Nutr Food Res.

[43] Blumensatt M, Greulich S, Herzfeld de Wiza D, Mueller H, Maxhera B, Rabelink MJ (2013). Activin A impairs insulin action in cardiomyocytes via up-regulation of miR-143. Cardiovasc Res..

[44] Chen WJ, Greulich S, van der Meer RW, Rijzewijk LJ, Lamb HJ, de Roos A (2013). Activin A is associated with impaired myocardial glucose metabolism and left ventricular remodeling in patients with uncomplicated type 2 diabetes. Cardiovasc Diabetol.

[45] Ye Y, Bajaj M, Yang HC, Perez-Polo JR, Birnbaum Y (2017). SGLT-2 inhibition with dapagliflozin reduces the activation of the Nlrp3/ASC inflammasome and attenuates the development of diabetic cardiomyopathy in mice with type 2 diabetes. Further augmentation of the effects with saxagliptin, a DPP4 inhibitor. Cardiovasc Drugs Ther..

[46] Yang F, Qin Y, Wang Y, Meng S, Xian H, Che H (2019). Metformin inhibits the NLRP3 inflammasome via AMPK/mTOR-dependent effects in diabetic cardiomyopathy. Int J Biol Sci.

[47] Song S, Ding Y, Dai GL, Zhang Y, Xu MT, Shen JR (2021). Sirtuin 3 deficiency exacerbates diabetic cardiomyopathy via necroptosis enhancement and NLRP3 activation. Acta Pharmacol Sin.

[48] Zhang H, Chen X, Zong B, Yuan H, Wang Z, Wei Y (2018). Gypenosides improve diabetic cardiomyopathy by inhibiting ROS-mediated NLRP3 inflammasome activation. J Cell Mol Med.

[49] Yao J, Li Y, Jin Y, Chen Y, Tian L, He W (2021). Synergistic cardioptotection by tilianin and syringin in diabetic cardiomyopathy involves interaction of TLR4/NF-kappaB/NLRP3 and PGC1a/SIRT3 pathways. Int Immunopharmacol.

[50] Hong L, Zha Y, Wang C, Qiao S, An J (2021). Folic acid alleviates high glucose and fat-induced pyroptosis via inhibition of the hippo signal pathway on H9C2 cells. Front Mol Biosci.

[51] Wang Y, Li H, Li Y, Zhao Y, Xiong F, Liu Y (2019). Coriolus versicolor alleviates diabetic cardiomyopathy by inhibiting cardiac fibrosis and NLRP3 inflammasome activation. Phytother Res.

[52] Luo B, Li B, Wang W, Liu X, Xia Y, Zhang C (2014). NLRP3 gene silencing ameliorates diabetic cardiomyopathy in a type 2 diabetes rat model. PLoS ONE.

[53] Luo B, Li B, Wang W, Liu X, Liu X, Xia Y (2014). Rosuvastatin alleviates diabetic cardiomyopathy by inhibiting NLRP3 inflammasome and MAPK pathways in a type 2 diabetes rat model. Cardiovasc Drugs Ther.

[54] Tektonidou MG, Papassotiriou I, Sfikakis PP (2021). Growth differentiation factor 15 (GDF-15) as potential cardiovascular risk biomarker in antiphospholipid syndrome. Rheumatology.

[55] Dominguez-Rodriguez A, Abreu-Gonzalez P, Avanzas P (2014). Usefulness of growth differentiation factor-15 levels to predict diabetic cardiomyopathy in asymptomatic patients with type 2 diabetes mellitus. Am J Cardiol.

[56] Uysal C, Arslan D, Buyukinan M, Gederet YT, Vatansev H, Ozcelik HS (2021). Growth differentiation factor-15 level and tissue doppler echocardiography as a tool in identification of cardiac effects in the children with type 1 diabetes mellitus. Exp Clin Endocrinol Diabetes..

[57] Lai S, Fu X, Yang S, Zhang S, Lin Q, Zhang M (2020). G protein-coupled receptor kinase-2: a potential biomarker for early diabetic cardiomyopathy. J Diabetes.

[58] Han Y, Lai J, Tao J, Tai Y, Zhou W, Guo P (2020). Sustaining circulating regulatory T cell subset contributes to the therapeutic effect of paroxetine on mice with diabetic cardiomyopathy. Circ J.

[59] Cipolletta E, Gambardella J, Fiordelisi A, Del Giudice C, Di Vaia E, Ciccarelli M (2019). Antidiabetic and cardioprotective effects of pharmacological inhibition of GRK2 in db/db mice. Int J Mol Sci..

[60] Wang Q, Liu Y, Fu Q, Xu B, Zhang Y, Kim S (2017). Inhibiting insulin-mediated beta2-adrenergic receptor activation prevents diabetes-associated cardiac dysfunction. Circulation.

[61] Yu XY, Chen HM, Liang JL, Lin QX, Tan HH, Fu YH (2011). Hyperglycemic myocardial damage is mediated by proinflammatory cytokine: macrophage migration inhibitory factor. PLoS ONE.

[62] Larsson B, Phillips SC (1998). Isolation and characterization of a novel, human neuronal nitric oxide synthase cDNA. Biochem Biophys Res Commun.

[63] Barnes G, Japp AG, Newby DE (2010). Translational promise of the apelin–APJ system. Heart.

[64] Barnes GD, Alam S, Carter G, Pedersen CM, Lee KM, Hubbard TJ (2013). Sustained cardiovascular actions of APJ agonism during renin-angiotensin system activation and in patients with heart failure. Circ Heart Fail.

[65] Szokodi I, Tavi P, Foldes G, Voutilainen-Myllyla S, Ilves M, Tokola H (2002). Apelin, the novel endogenous ligand of the orphan receptor APJ, regulates cardiac contractility. Circul Res.

[66] Maguire JJ, Kleinz MJ, Pitkin SL, Davenport AP (2009). [Pyr1]apelin-13 identified as the predominant apelin isoform in the human heart: vasoactive mechanisms and inotropic action in disease. Hypertension.

[67] Wang C, Du JF, Wu F, Wang HC (2008). Apelin decreases the SR Ca2+ content but enhances the amplitude of [Ca2+]i transient and contractions during twitches in isolated rat cardiac myocytes. Am J Physiol Heart Circ Physiol.

[68] Falcao-Pires I, Goncalves N, Gavina C, Pinho S, Teixeira T, Moura C (2010). Correlation between plasma levels of apelin and myocardial hypertrophy in rats and humans: possible target for treatment?. Expert Opin Ther Targets.

[69] Japp AG, Cruden NL, Amer DA, Li VK, Goudie EB, Johnston NR (2008). Vascular effects of apelin in vivo in man. J Am Coll Cardiol.

[70] Schinzari F, Veneziani A, Mores N, Barini A, Di Daniele N, Cardillo C (2017). Beneficial effects of apelin on vascular function in patients with central obesity. Hypertension.

[71] Li B, Yin J, Chang J, Zhang J, Wang Y, Huang H (2021). Apelin/APJ relieve diabetic cardiomyopathy by reducing microvascular dysfunction. J Endocrinol.

[72] Zeng H, He X, Hou X, Li L, Chen JX (2014). Apelin gene therapy increases myocardial vascular density and ameliorates diabetic cardiomyopathy via upregulation of sirtuin 3. Am J Physiol Heart Circ Physiol.

[73] Alfarano C, Foussal C, Lairez O, Calise D, Attane C, Anesia R (2015). Transition from metabolic adaptation to maladaptation of the heart in obesity: role of apelin. Int J Obes (Lond).

[74] Ansari A, Rahman MS, Saha SK, Saikot FK, Deep A, Kim KH (2017). Function of the SIRT3 mitochondrial deacetylase in cellular physiology, cancer, and neurodegenerative disease. Aging Cell..

[75] Ni T, Lin N, Huang X, Lu W, Sun Z, Zhang J (2020). Icariin ameliorates diabetic cardiomyopathy through Apelin/Sirt3 signalling to improve mitochondrial dysfunction. Front Pharmacol.

[76] Li L, Zeng H, Hou X, He X, Chen JX (2013). Myocardial injection of apelin-overexpressing bone marrow cells improves cardiac repair via upregulation of Sirt3 after myocardial infarction. PLoS ONE.

[77] Hou X, Zeng H, Tuo QH, Liao DF, Chen JX (2015). Apelin gene therapy increases autophagy via activation of Sirtuin 3 in diabetic heart. Diabetes Res.

[78] Hou X, Zeng H, He X, Chen JX (2015). Sirt3 is essential for apelin-induced angiogenesis in post-myocardial infarction of diabetes. J Cell Mol Med.

[79] Siddiquee K, Hampton J, Khan S, Zadory D, Gleaves L, Vaughan DE (2011). Apelin protects against angiotensin II-induced cardiovascular fibrosis and decreases plasminogen activator inhibitor type-1 production. J Hypertens.

[80] Wang W, Zhang D, Yang R, Xia W, Qian K, Shi Z (2018). Hepatic and cardiac beneficial effects of a long-acting Fc-apelin fusion protein in diet-induced obese mice. Diabetes Metab Res Rev.

[81] Lv W, Zhang L, Cheng X, Wang H, Qin W, Zhou X (2020). Apelin Inhibits Angiotensin II-Induced Atrial Fibrosis and Atrial Fibrillation via TGF-beta1/Smad2/alpha-SMA Pathway. Front Physiol.

[82] Zhong S, Guo H, Wang H, Xing D, Lu T, Yang J (2020). Apelin-13 alleviated cardiac fibrosis via inhibiting the PI3K/Akt pathway to attenuate oxidative stress in rats with myocardial infarction-induced heart failure. Biosci Rep..

[83] Verma S, Eikelboom JW, Nidorf SM, Al-Omran M, Gupta N, Teoh H (2015). Colchicine in cardiac disease: a systematic review and meta-analysis of randomized controlled trials. BMC Cardiovasc Disord.

[84] Akbal E, Ozbek M, Gunes F, Akyurek O, Ureten K, Delibasi T (2009). Serum heart type fatty acid binding protein levels in metabolic syndrome. Endocrine.

[85] Shearer J, Fueger PT, Wang Z, Bracy DP, Wasserman DH, Rottman JN (2008). Metabolic implications of reduced heart-type fatty acid binding protein in insulin resistant cardiac muscle. Biochim Biophys Acta.

[86] Ramesh P, Chauhan A, Goyal P, Singh A, Singhal A, Gupta A (2020). Serum heart type fatty acid binding protein levels in prediabetes-an invaluable cardiovascular biomarker. J Assoc Phys India.

[87] Akbal A, Kurtaran A, Selcuk B, Akyuz M (2013). H-FABP, cardiovascular risk factors, and functional status in asymptomatic spinal cord injury patients. Herz.

[88] Alabi TD, Chegou NN, Brooks NL, Oguntibeju OO (2020). Effects of anchomanes difformis on inflammation, apoptosis, and organ toxicity in STZ-induced diabetic cardiomyopathy. Biomedicines..

[89] Ajiboye BO, Oyinloye BE, Onikanni SA, Osukoya OA, Lawal OE, Bamisaye FA (2021). *Sterculia*
*tragacantha* Lindl aqueous leaf extract ameliorate cardiomyopathy in streptozotocin-induced diabetic rats via urotensin II and FABP3 expressions. J Oleo Sci.

[90] Li W, Yao M, Wang R, Shi Y, Hou L, Hou Z (2018). Profile of cardiac lipid metabolism in STZ-induced diabetic mice. Lipids Health Dis.

[91] Suthahar N, Meijers WC, Sillje HHW, Ho JE, Liu FT, de Boer RA (2018). Galectin-3 activation and inhibition in heart failure and cardiovascular disease: an update. Theranostics..

[92] Travers JG, Kamal FA, Robbins J, Yutzey KE, Blaxall BC (2016). Cardiac fibrosis: the fibroblast awakens. Circul Res.

[93] Martinez-Martinez E, Calvier L, Fernandez-Celis A, Rousseau E, Jurado-Lopez R, Rossoni LV (2015). Galectin-3 blockade inhibits cardiac inflammation and fibrosis in experimental hyperaldosteronism and hypertension. Hypertension.

[94] Luis C, Costa R, Rodrigues I, Castela A, Coelho P, Guerreiro S (2019). Xanthohumol and 8-prenylnaringenin reduce type 2 diabetes-associated oxidative stress by downregulating galectin-3. Porto Biomed J.

[95] Azibani F, Benard L, Schlossarek S, Merval R, Tournoux F, Fazal L (2012). Aldosterone inhibits antifibrotic factors in mouse hypertensive heart. Hypertension.

[96] Ibarrola J, Sadaba R, Garcia-Pena A, Arrieta V, Martinez-Martinez E, Alvarez V (2018). A role for fumarate hydratase in mediating oxidative effects of galectin-3 in human cardiac fibroblasts. Int J Cardiol.

[97] Lebedev DA, Lyasnikova EA, Vasilyeva EY, Likhonosov NP, Sitnikova MY, Babenko AY (2021). Association between markers of fibrosis and heart failure incidence in patients with type 2 diabetes mellitus. J Diabetes Res.

[98] Holmager P, Egstrup M, Gustafsson I, Schou M, Dahl JS, Rasmussen LM (2017). Galectin-3 and fibulin-1 in systolic heart failure - relation to glucose metabolism and left ventricular contractile reserve. BMC Cardiovasc Disord.

[99] Flores-Ramirez R, Azpiri-Lopez JR, Gonzalez-Gonzalez JG, Ordaz-Farias A, Gonzalez-Carrillo LE, Carrizales-Sepulveda EF (2017). Global longitudinal strain as a biomarker in diabetic cardiomyopathy. A comparative study with Gal-3 in patients with preserved ejection fraction. Arch Cardiol Mex..

[100] Gruson D, Mancini M, Ahn SA, Rousseau MF (2014). Galectin-3 testing: validity of a novel automated assay in heart failure patients with reduced ejection fraction. Clin Chim Acta.

[101] Ho JE, Liu C, Lyass A, Courchesne P, Pencina MJ, Vasan RS (2012). Galectin-3, a marker of cardiac fibrosis, predicts incident heart failure in the community. J Am Coll Cardiol.

[102] Gopal DM, Ayalon N, Wang YC, Siwik D, Sverdlov A, Donohue C (2019). Galectin-3 is associated with stage B metabolic heart disease and pulmonary hypertension in young obese patients. J Am Heart Assoc.

[103] Yingchoncharoen T, Agarwal S, Popovic ZB, Marwick TH (2013). Normal ranges of left ventricular strain: a meta-analysis. J Am Soc Echocardiogr.

[104] Nayor M, Wang N, Larson MG, Vasan RS, Levy D, Ho JE (2015). Circulating galectin-3 is associated with cardiometabolic disease in the community. J Am Heart Assoc..

[105] Wu X, Liu Y, Tu D, Liu X, Niu S, Suo Y (2020). Role of NLRP3-inflammasome/caspase-1/galectin-3 pathway on atrial remodeling in diabetic rabbits. J Cardiovasc Transl Res.

[106] Seferovic JP, Lalic NM, Floridi F, Tesic M, Seferovic PM, Giga V (2014). Structural myocardial alterations in diabetes and hypertension: the role of galectin-3. Clin Chem Lab Med.

[107] Xu GR, Zhang C, Yang HX, Sun JH, Zhang Y, Yao TT (2020). Modified citrus pectin ameliorates myocardial fibrosis and inflammation via suppressing galectin-3 and TLR4/MyD88/NF-kappaB signaling pathway. Biomed Pharmacother.

[108] Lepojarvi ES, Piira OP, Paakko E, Lammentausta E, Risteli J, Miettinen JA (2015). Serum PINP, PIIINP, galectin-3, and ST2 as surrogates of myocardial fibrosis and echocardiographic left venticular diastolic filling properties. Front Physiol.

[109] Nguyen MN, Ziemann M, Kiriazis H, Su Y, Thomas Z, Lu Q (2019). Galectin-3 deficiency ameliorates fibrosis and remodeling in dilated cardiomyopathy mice with enhanced Mst1 signaling. Am J Physiol Heart Circ Physiol.

[110] Zhu N, Zhu L, Huang B, Xiang W, Zhao X (2022). Galectin-3 inhibition ameliorates streptozotocin-induced diabetic cardiomyopathy in mice. Front Cardiovasc Med.

[111] Pennica D, King KL, Shaw KJ, Luis E, Rullamas J, Luoh SM (1995). Expression cloning of cardiotrophin 1, a cytokine that induces cardiac myocyte hypertrophy. Proc Natl Acad Sci USA.

[112] Asai S, Saito Y, Kuwahara K, Mizuno Y, Yoshimura M, Higashikubo C (2000). The heart is a source of circulating cardiotrophin-1 in humans. Biochem Biophys Res Commun.

[113] Kumric M, Ticinovic Kurir T, Borovac JA, Bozic J (2021). Role of novel biomarkers in diabetic cardiomyopathy. World J Diabetes.

[114] Garcia-Cenador MB, Lopez-Novoa JM, Diez J, Garcia-Criado FJ (2013). Effects and mechanism of organ protection by cardiotrophin-1. Curr Med Chem.

[115] Robador PA, San Jose G, Rodriguez C, Guadall A, Moreno MU, Beaumont J (2011). HIF-1-mediated up-regulation of cardiotrophin-1 is involved in the survival response of cardiomyocytes to hypoxia. Cardiovasc Res.

[116] Ravassa S, Beloqui O, Varo N, Barba J, Lopez B, Beaumont J (2013). Association of cardiotrophin-1 with left ventricular systolic properties in asymptomatic hypertensive patients. J Hypertens.

[117] Monserrat L, Lopez B, Gonzalez A, Hermida M, Fernandez X, Ortiz M (2011). Cardiotrophin-1 plasma levels are associated with the severity of hypertrophy in hypertrophic cardiomyopathy. Eur Heart J.

[118] Ruiz-Hurtado G, Gomez-Hurtado N, Fernandez-Velasco M, Calderon E, Smani T, Ordonez A (2012). Cardiotrophin-1 induces sarcoplasmic reticulum Ca(2+) leak and arrhythmogenesis in adult rat ventricular myocytes. Cardiovasc Res.

[119] Freed DH, Chilton L, Li Y, Dangerfield AL, Raizman JE, Rattan SG (2011). Role of myosin light chain kinase in cardiotrophin-1-induced cardiac myofibroblast cell migration. Am J Physiol Heart Circ Physiol.

[120] Moreno-Aliaga MJ, Perez-Echarri N, Marcos-Gomez B, Larequi E, Gil-Bea FJ, Viollet B (2011). Cardiotrophin-1 is a key regulator of glucose and lipid metabolism. Cell Metab.

[121] Liu J, Liu Z, Huang F, Xing Z, Wang H, Li Z (2007). Pioglitazone inhibits hypertrophy induced by high glucose and insulin in cultured neonatal rat cardiomyocytes. Pharmazie.

[122] El-Saiedi SA, Hafez MH, Sedky YM, Sharaf SA, Kamel MS, AbdelMassih AF (2021). Novel biomarkers for subtle myocardial involvement in type I diabetes mellitus. Cardiovasc Endocrinol Metab.

[123] Gamella-Pozuelo L, Fuentes-Calvo I, Gomez-Marcos MA, Recio-Rodriguez JI, Agudo-Conde C, Fernandez-Martin JL (2015). Plasma cardiotrophin-1 as a marker of hypertension and diabetes-induced target organ damage and cardiovascular risk. Medicine.

[124] Tsutamoto T, Wada A, Maeda K, Mabuchi N, Hayashi M, Tsutsui T (2001). Relationship between plasma level of cardiotrophin-1 and left ventricular mass index in patients with dilated cardiomyopathy. J Am Coll Cardiol.

[125] Briana DD, Germanou K, Boutsikou M, Boutsikou T, Athanasopoulos N, Marmarinos A (2018). Potential prognostic biomarkers of cardiovascular disease in fetal macrosomia: the impact of gestational diabetes. J Matern Fetal Neonatal Med.

[126] Jimenez-Gonzalez M, Jaques F, Rodriguez S, Porciuncula A, Principe RM, Abizanda G (2013). Cardiotrophin 1 protects beta cells from apoptosis and prevents streptozotocin-induced diabetes in a mouse model. Diabetologia.

[127] Jin H, Yang R, Ko A, Pennica D, Wood WI, Paoni NF (1998). Effects of cardiotrophin-1 on haemodynamics and cardiac function in conscious rats. Cytokine.

[128] Vepsalainen T, Soinio M, Marniemi J, Lehto S, Juutilainen A, Laakso M (2011). Physical activity, high-sensitivity C-reactive protein, and total and cardiovascular disease mortality in type 2 diabetes. Diabetes Care.

[129] Cui X, Chopp M, Zacharek A, Ye X, Roberts C, Chen J (2011). Angiopoietin/Tie2 pathway mediates type 2 diabetes induced vascular damage after cerebral stroke. Neurobiol Dis.

[130] Rosin NL, Falkenham A, Sopel MJ, Lee TD, Legare JF (2013). Regulation and role of connective tissue growth factor in AngII-induced myocardial fibrosis. Am J Pathol.

[131] Daniels A, van Bilsen M, Goldschmeding R, van der Vusse GJ, van Nieuwenhoven FA (2009). Connective tissue growth factor and cardiac fibrosis. Acta Physiol (Oxf).

[132] Bayerle-Eder M, Zangeneh M, Kreiner G, Raffesberg W, Nowotny P, Vierhapper H (2003). ANP but not BNP reflects early left diastolic dysfunction in type 1 diabetics with myocardial dysinnervation. Horm Metab Res.

[133] Nishikimi T, Maeda N, Matsuoka H (2006). The role of natriuretic peptides in cardioprotection. Cardiovasc Res.

[134] Masuda T, Muto S, Fujisawa G, Iwazu Y, Kimura M, Kobayashi T (2012). Heart angiotensin II-induced cardiomyocyte hypertrophy suppresses coronary angiogenesis and progresses diabetic cardiomyopathy. Am J Physiol Heart Circ Physiol.

[135] Kajstura J, Fiordaliso F, Andreoli AM, Li B, Chimenti S, Medow MS (2001). IGF-1 overexpression inhibits the development of diabetic cardiomyopathy and angiotensin II-mediated oxidative stress. Diabetes.

[136] Vague P, Coste TC, Jannot MF, Raccah D, Tsimaratos M (2004). C-peptide, Na+, K(+)-ATPase, and diabetes. Exp Diabesity Res.

[137] Korkmaz-Icoz S, Lehner A, Li S, Vater A, Radovits T, Brune M (2016). Left ventricular pressure-volume measurements and myocardial gene expression profile in type 2 diabetic Goto-Kakizaki rats. Am J Physiol Heart Circ Physiol.

[138] Russell NE, Higgins MF, Amaruso M, Foley M, McAuliffe FM (2009). Troponin T and pro-B-type natriuretic peptide in fetuses of type 1 diabetic mothers. Diabetes Care.

[139] Korraa A, Ezzat MH, Bastawy M, Aly H, El-Mazary AA, Abd E-A (2012). Cardiac troponin I levels and its relation to echocardiographic findings in infants of diabetic mothers. Ital J Pediatr.

[140] Al-Rasheed NM, Al-Rasheed NM, Hasan IH, Al-Amin MA, Al-Ajmi HN, Mohamad RA (2017). Simvastatin ameliorates diabetic cardiomyopathy by attenuating oxidative stress and inflammation in rats. Oxid Med Cell Longev.

[141] Janghorbani M, Amini M (2009). Progression to impaired glucose metabolism in first-degree relatives of patients with type 2 diabetes in Isfahan. Iran Diabetes Metab Res Rev.

[142] Liu Z, Zhao N, Zhu H, Zhu S, Pan S, Xu J (2015). Circulating interleukin-1beta promotes endoplasmic reticulum stress-induced myocytes apoptosis in diabetic cardiomyopathy via interleukin-1 receptor-associated kinase-2. Cardiovasc Diabetol.

[143] Abukhalil MH, Althunibat OY, Aladaileh SH, Al-Amarat W, Obeidat HM, Al-Khawalde AAA (2021). Galangin attenuates diabetic cardiomyopathy through modulating oxidative stress, inflammation and apoptosis in rats. Biomed Pharmacother.

[144] Sun M, Chen M, Dawood F, Zurawska U, Li JY, Parker T (2007). Tumor necrosis factor-alpha mediates cardiac remodeling and ventricular dysfunction after pressure overload state. Circulation.

[145] Cividini F, Scott BT, Dai A, Han W, Suarez J, Diaz-Juarez J (2016). O-GlcNAcylation of 8-oxoguanine DNA glycosylase (Ogg1) impairs oxidative mitochondrial DNA lesion repair in diabetic hearts. J Biol Chem.

[146] Ban CR, Twigg SM, Franjic B, Brooks BA, Celermajer D, Yue DK (2010). Serum MMP-7 is increased in diabetic renal disease and diabetic diastolic dysfunction. Diabetes Res Clin Pract.

[147] Van Linthout S, Seeland U, Riad A, Eckhardt O, Hohl M, Dhayat N (2008). Reduced MMP-2 activity contributes to cardiac fibrosis in experimental diabetic cardiomyopathy. Basic Res Cardiol.

[148] Lopez-Andres N, Fortuno MA, Diez J, Zannad F, Lacolley P, Rossignol P (2010). Vascular effects of cardiotrophin-1: a role in hypertension?. J Hypertens.

